# γ sulphate PNA (PNA S): Highly Selective DNA Binding Molecule Showing Promising Antigene Activity

**DOI:** 10.1371/journal.pone.0035774

**Published:** 2012-05-07

**Authors:** Concetta Avitabile, Loredana Moggio, Gaetano Malgieri, Domenica Capasso, Sonia Di Gaetano, Michele Saviano, Carlo Pedone, Alessandra Romanelli

**Affiliations:** 1 Dipartimento delle Scienze Biologiche, Facoltà di Scienze Biotecnologiche, Università di Napoli “Federico II”, Napoli, Italy; 2 Dipartimento di Scienze Ambientali, Seconda Università degli Studi di Napoli, Caserta, Italy; 3 Istituto di Biostrutture e Bioimmagini (CNR), Napoli, Italy; 4 Istituto di Cristallografia (CNR), Bari (Italy); University of Helsinki, Finland

## Abstract

Peptide Nucleic Acids (PNAs), nucleic acid analogues showing high stability to enzyme degradation and strong affinity and specificity of binding toward DNA and RNA are widely investigated as tools to interfere in gene expression. Several studies have been focused on PNA analogues with modifications on the backbone and bases in the attempt to overcome solubility, uptake and aggregation issues. γ PNAs, PNA derivatives having a substituent in the γ position of the backbone show interesting properties in terms of secondary structure and affinity of binding toward complementary nucleic acids. In this paper we illustrate our results obtained on new analogues, bearing a sulphate in the γ position of the backbone, developed to be more DNA-like in terms of polarity and charge. The synthesis of monomers and oligomers is described. NMR studies on the conformational properties of monomers and studies on the secondary structure of single strands and triplexes are reported. Furthermore the hybrid stability and the effect of mismatches on the stability have also been investigated. Finally, the ability of the new analogue to work as antigene, interfering with the transcription of the ErbB2 gene on a human cell line overexpressing ErbB2 (SKBR3), assessed by FACS and qPCR, is described.

## Introduction

The search for stable and biologically active DNA mimics has encouraged studies on Peptide Nucleic Acids (PNAs), nucleic acid analogues showing high stability to enzyme degradation and strong affinity and specificity of binding toward DNA and RNA. [1], [2], [3], [4] Several PNA analogues with modification on the backbone and bases have been obtained so far in the attempt to overcome solubility, uptake and aggregation issues. [5], [6], [7], [8], [9], [10], [11], [12], [13], [14], [15], [16], [17], [18], [19], [20], [21], [22], [23], [24], [25] GPNAs, PNA analogues with a guanidine group in the α position of the backbone, designed to target the transcriptional start-site of the E-cadherin gene, were demonstrated to effectively inhibit protein translation, showing low toxicity and high cell permeability. [5] These analogues were found unable to inhibit gene transcription, probably due to electrostatic interactions with the phosphates of nucleic acids, which slow down the diffusion of the molecules in the cytoplasm and in the nucleus. [5] Studies on constrained PNA oligomers showing clear conformational preferences have demonstrated that preorganization is a requisite to increase the affinity of binding toward complementary nucleic acids. [11], [12] A recently investigated family of PNA analogues is represented by γ Peptide Nucleic Acid (γ PNA), PNA derivatives bearing a substituent, usually corresponding to the side chain of a natural amino acid, in the gamma position of the backbone. Several analogues have been explored so far, having methyl, hydroxymethyl, thiomethyl, aminobutyl and guanidinium groups attached. [13], [14], [15], [16], [17], [18], [19], [20], [21], [22], [23], [24] CD studies on PNA oligomers containing γ hydroxymethyl have shown that these oligomers are structurally preorganized; PNA oligomers containing (S) γ Ser-PNA units show spectra typical of right handed PNA-DNA duplexes while PNAs containing (R) γ Ser-PNA form left handed helices. [19] The secondary structure of the oligomers depends on the position of the chiral moiety and is induced when the chiral moiety is at the C-terminus. Recently the crystal structure of a γ PNA/DNA duplex has been solved; these studies revealed that the helix is induced by the steric clash between the γ carbon and the nitrogen of the tertiary amide of the backbone and is stabilized by the sequential base stacking. [26] The incorporation of one S chiral unit in a PNA oligomer enhances the stability of the duplex hybrids, yielding an increase of 3°C in the melting temperature of PNA/DNA duplexes and of 2°C for PNA/RNA hybrids; [19] oligomers containing monomers with R chirality, instead, bind DNA and RNA with low affinity and selectivity. Interestingly the stability of the hybrids is not influenced by the spacing between the chiral units. Furthermore γ PNA is the only analogue discovered so far having the ability to invade a mixed sequence DNA double helix. [27].

Encouraged by these results and with the aim to develop a new PNA analogue more DNA-like in terms of polarity, charge and solubility we undertook studies on γ PNAs having a sulphate moiety in the γ position of the backbone. The sulphate group is, in fact, very similar to the phosphate of DNA, in geometry, steric hindrance, polarity; furthermore a sulphate monoester has the same charge as the phosphate diester present in oligonucleotides. The sulphate PNAs should in principle keep the advantages of standard PNAs (stability to degradation and binding affinity) and in addition should be more soluble, less prone to aggregation. Two more advantages of this negatively charged analogue should be: 1) the lack of unspecific interactions with nucleic acids, which instead may occur with positively charged PNA analogues, as observed in α guanidine PNA; 2) the possibility to increase its uptake employing the tools commonly used for DNA transfection. In fact PNA and PNA analogues (with the only exception of γ guanidine PNA), [23] although showing high binding affinity towards natural nucleic acids and high stability to enzymatic degradation, do not easily cross the cell membrane and consequently show weak biological activity. In this respect, it is worth noting that many studies have been carried out with the aim to improve the uptake of PNA; the delivery of PNA by lipofectamine or other cationic lipids employed for the common DNA delivery has been demonstrated only for PNA complexed to DNA or PNA conjugated to negatively charged peptides. [28], [29], [30], [31], [32], [33] To evaluate the antigene activity of sulphate PNA, the *ErbB2* gene was chosen as target. *ErbB2* is a known proto-oncogene over-expressed in numerous cancers, including breast and ovarian tumors and is correlated to increased chemoresistance of the cancer cells. [34] *ErbB2* encodes a protein member of the EGFR/ErbB protein family and is a cell membrane surface-bound receptor tyrosine kinase, which is normally involved in the signal transduction pathways leading to cell growth and differentiation. [35] EGFR signaling is activated by unspecific ligand binding to the extracellular domain of the receptor. This determines receptor homo−/hetero-dimerization and a subsequent autophosphorylation by the intracellular kinase domain, resulting in receptor activation. After activation, phosphorylation of cytoplasmic substrates occurs and a signaling cascade drives many cellular responses, which include changes in gene expression, cytoskeleton rearrangement, anti-apoptosis and increased cell proliferation. [36].

In this work we investigated γ sulphate PNAs. We set up protocols for the synthesis of the four PNA monomers having the sulphate group in the γ position of the backbone and of oligomers containing sulphate monomers. The conformational preferences of the PNA monomers were investigated by NMR. Studies on the secondary structure of a polypirimidine oligomer were carried out by CD. The ability of the modified oligomer to interact with DNA, the specificity and affinity of binding were investigated by UV and CD. Finally, we explored the ability of the sulphate PNA to interfere with the transcription of the *ErbB2* gene by targeting its promoter on a human cell line overexpressing ErbB2 (SKBR3). The sequence chosen as target is localized from −220 to −228 upstream of the first codon. [37] Analysis performed by FACS, fluorescence microscopy and qPCR, demonstrated that PNA S inhibits gene expression of HER-2 and reduces ErbB2 receptor number on cell surface.

## Results

### Synthesis of the PNA Monomers and Oligomers

**Figure 1:**
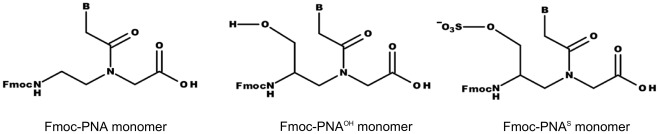
PNA monomers. Schematic representation of standard and modified PNA monomers.

The PNA γ sulphate monomers are obtained after derivatization of γ hydroxymethyl monomers (Figure 1,), synthesized using a modified version of the protocol reported in the literature for γ hydroxymethyl PNA starting from L-Fmoc-Ser(OtBu)-OH. [19] (Supporting S1).

In the monomers the exocyclic amines of the nucleobases A, C and G are protected with base labile groups, benzoyl for A and C, isobutyryl for G. [38], [39], [40] The sulphate was installed on the hydroxyl after reaction with the DMF•SO_3_ complex and treatment with tetrabutylammonium (TBA) hydrogen sulfate/NaHCO_3_. The treatment of the sulphate monomers with tetrabutylammonium is needed in order to stabilize the acid labile sulphate group. [41] The stability of the sulphate to both acidic and basic conditions was verified in two separate experiments, carried out treating the t^S^ monomer with the cleavage and deprotection solutions, respectively a concentrated TFA solution for 2 hours at room temperature and a concentrated ammonia solution at 50°C for 16 hours (data not shown). The reactions were monitored by LC-MS; except for the removal of the Fmoc group occurring in basic conditions, no other reaction was observed, demonstrating thus that the TBA counterion is sufficient to stabilize the sulphate group. All monomers were characterized by electrospray mass analysis and NMR.

The coupling conditions on solid phase for all the monomers were set up testing different mixtures of activators and bases. The best coupling conditions are found to be the following. For Fmoc- t^S^-OH: 50 µL of a 0.3 M solution (7.9 eq.) in an. DMF of monomer, 50 µL of HBTU (0.2 M) (5.2 eq.) in DMF, 50 µL MDCH (0.8 M) in pyridine, 30 minutes. For Fmoc- c^S^ (Bz)-OH, Fmoc-a^S^ (Bz)-OH, Fmoc g^S^ (iBu)-OH: 50 µL of a 0.3 M solution (7.9 eq.) in an. DMF of monomer, 50 µL of HBTU (0.2 M) (5.2 eq.) in DMF, NMM 0.2 M, 50 µL of pyridine 0.2 M in DMF, 30 minutes.

The sequence chosen to target the *ErbB2* gene promoter, CTCCTCCTC, encompasses the tract from −220 to −228 upstream of the first codon. [37] In the sequence all T were inserted as sulphate T (t^S^).

The oligomer ct^S^cct^S^cct^S^c (PNA S) was assembled on a PAL-PEG resin using standard conditions for the standard monomers and the conditions described earlier for the t^S^ building blocks. The oligomer was cleaved in standard conditions, purified by RP-HPLC and characterized by LC-MS. (Supporting S2 and Figure S1).

The unmodified PNA ctcctcctc (PNA) was obtained using standard protocols, [42] purified by RP-HPLC, characterized by electrospray and used in the control experiments. (Supporting S2).

The FITC conjugated PNA S and PNA were obtained coupling at the N-terminus of PNA S or PNA first the amino-hexanoic acid linker and then the FITC. (Supporting S3, Figure S2 and Figure S3).

The oligomer containing hydroxymethyl modified γ PNA T monomer (t^OH^), c t^OH^cct^OH^cct^OH^c (PNA OH) was obtained as the ct^S^cct^S^cct^S^c and the t^OH^ monomer was coupled as the t^S^ monomer. (Supporting S2).

### NMR Studies

We performed NMR experiments on Fmoc-PNA ^OH^ and Fmoc- PNA^S^ monomers dissolved in DMSO.

We will describe results obtained for the guanine monomer, but the same spectral features were also observed for the other monomers.

**Figure 2:**
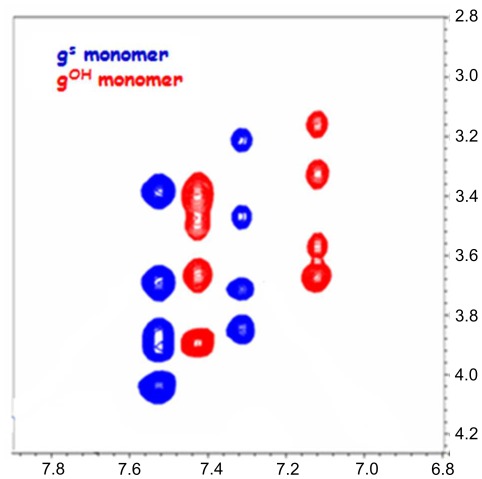
NMR characterization of the monomers. Superimposition of the 2D-TOCSY, expansion of the backbone region, for the g^S^ and g^OH^ monomers.

An expansion of the backbone region of the 2D-TOCSY for the two guanine monomers (g^OH^ in red and g^S^-OH in blue) is reported in Figure 2.

Because of the well-known restricted rotation around the tertiary amide bond in PNA that produces two possible rotamers in solution around the C7–N4 bond (tertiary amide bond), the spin systems in the NMR spectrum are duplicated. As expected, the introduction of the sulphate group in the γ position of the backbone results in a discernible shift of the signals toward lower fields (Table 1).

**Table 1:** ^1^H chemical shift (ppm) for the g^S^ and g^OH^ backbone protons. See Figure 3 for numbering.

	g^S^ rotamer A	g^S^ rotamer B	g^OH^ rotamer A	g^OH^ rotamer B
1	7.53	7.32	7.43	7.12
2	4.04	3.85	3.90	3.67
3a	3.69	3.47	3.49	3.33
3b	3.39	3.21	3.37	3.16
9	3.87	3.71	3.67	3.57

**Figure 3:**
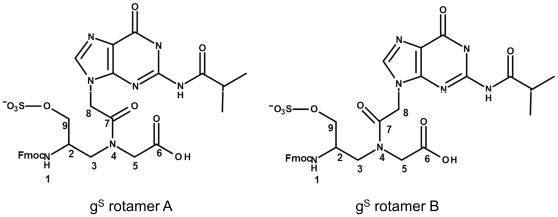
Monomers rotamers. Representation of the two rotamers found for the sulphate monomer g^S^.

**Figure 4:**
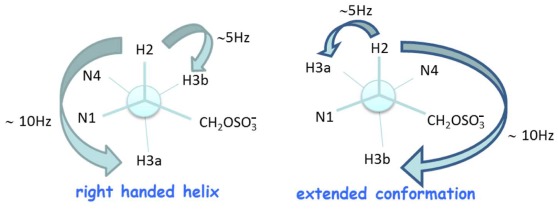
Sulphate monomers conformation. The two hypothesized conformations for the g^S^ monomers.

The NOESY spectrum has been used to determine the geometry of the tertiary amide bond: a NOE cross-correlation peak between H-8 and H-3 is found in the conformation where the carbonyl C-7 points toward the C terminus (rotamer A), and a NOE cross-correlation peak between H-8 and H-5 where the C-7 carbonyl group points toward the N terminus (rotamer B) (Figure 3). A clear difference is evident between the two monomers: the signals for rotamer A and B of the Fmoc-g^OH^-OH monomer have a comparable intensity while in the Fmoc-g^S^-OH monomer the signals of rotamer A have about two times the intensity of those of rotamer B. The conformational preferences of the Fmoc-g^S^-OH monomer have been deduced by identifying and evaluating the coupling pattern of the spin system composed of H-2, H-3a, H-3b in the DQ-COSY experiment. The signal of H-3a for rotamer A appears as a doublet of doublets with coupling constants of ∼15 and ∼10 Hz, while H-3b appears as a doublet of doublets with coupling constants of ∼15 and ∼5 Hz. This suggests the presence of a preferred conformation in solution that involves one trans diaxial interaction (180°) between H-2 and H-3a (J of ∼10 Hz) and an axial to equatorial interaction (+60°) between H-2 and H-3b (J of ∼5 Hz). The ∼15 Hz coupling constant corresponds to the geminal coupling (J2) between H-3a and H-3b. For rotamer B we observe: the ∼10 Hz coupling constant corresponding to a trans diaxial relationship measured between H-3b and H-2, and a ∼5 Hz coupling constant corresponding to an equatorial to axial relationship between H-3a and H-2. With R being the absolute configuration at C-2, the conformation that produces the relative spatial arrangement of protons H-2, H-3a, and H-3b in rotamer A corresponds to that observed in PNA monomers when they are in oligomers forming a right-handed helix. The conformation of rotamer B corresponds to an extended conformation. (Figure 4).

### Determination of the Stoichiometry of the Complexes by the Continuous Variation Method

Polypirimidine PNA sequences are known to hybridize DNA forming triplexes of stoichiometry PNA_2_/DNA. In order to determine the stoichiometry of the polypirimidine PNA S/DNA complex, we employed the continuous variation method. The absorbance at 260 nm of PNA S (and PNA) and DNA solutions at different PNA S (PNA)/DNA ratios was measured. The plots of the absorbance *vs* the molar fraction of the PNA S (PNA) show in both cases a minimum around 0.7 of molar fraction, which suggests a stoichiometry 2∶1 PNA S (PNA):DNA. The presence of sulphates in the side chain of the oligomer PNA S does not hamper the formation of hybrids with complementary DNA; the stoichiometry of the hybrid suggests that PNA S follows the same hybridization rules as standard PNA.

### CD Studies

**Figure 5:**
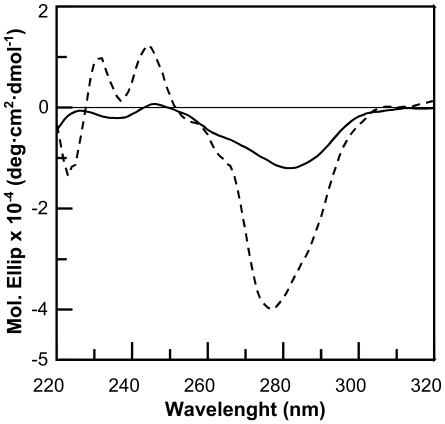
CD of single strands. CD spectra of PNA S (–) and PNA OH (–) in 10 mM Phosphate buffer 100 mM NaCl, 5 mM MgCl_2_, pH 7 at 10 µM strand concentration.

**Figure 6:**
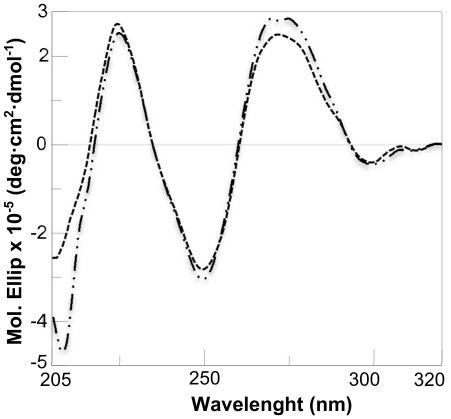
CD of triplexes. CD spectra of (PNA S)_2_DNAc (–), (PNA)_2_DNAc (–) in 10 mM Na Phosphate buffer, 100 mM NaCl, 5 mM MgCl_2_, pH 7 at 3 µM triplex concentration.

CD spectra of the oligomer ct^S^cct^S^cct^S^c (PNA S) were recorded at different concentrations. A very weak signal appears when the oligomer has a 10 µM concentration; the CD spectrum shows a deep minimum at 267 nm and a very shallow minimum at 238 nm. The CD spectrum of PNA S was compared to that of PNA OH, the analogue oligomer containing t^OH^ monomers (Table 2 and Figure 5): the spectrum of PNA OH shows more intense bands than the spectrum of PNA S. The structure of the triplexes formed by PNA S with complementary DNA was also investigated by CD and compared to the structure of a triplex formed by the unmodified PNA oligomer. CD spectra for the two hybrids are superimposable, showing two maxima at 270 and 225 nm and one minimum at 250 nm (Figure 6). The spectrum recorded for the DNA triplex, as expected, is different. PNA S forms a triple helix having the features of the P-helix formed by standard PNA. [43]

**Table 2:** Oligomers sequences. PNA bases are indicated with lower case letters, DNA bases with upper case letters.

Name	sequence	number
PNA S	ct^S^cct^S^cct^S^c	1
PNA	ctcctcctc	2
PNA OH	ct^OH^cct^OH^cct^OH^c	3
DNA	CTCCTCCTC	4
DNAc	GAGGAGGAG	5
DNA c mis	GAGCAGGAG	6
DNA c mis S	GAGGTGGAG	7

The stability of the hybrid was investigated by melting experiments followed by CD. Melting curves were recorded for the (PNA S)_2_DNAc and, as a control, for the (PNA)_2_DNAc and for the (DNA)_2_DNAc triplexes (see Table 2 for the sequences of the oligomers).

In all cases a single transition was observed. The Tm_s_ were estimated to be 52.2°C for (PNA)_2_DNAc, 46.6°C for (PNA S)_2_DNAc and 42.2°C for (DNA)_2_DNAc (Table 3). The effect of the introduction of one mismatch in the complementary DNA strand on the melting temperature of the hybrids was investigated in two cases: when the mismatched base is complementary to a PNA monomer in PNA S (DNA mis: GAGCAGGAG) and when the mismatched base is complementary to a t^S^ monomer (DNAc mis S: GAGGTGGAG). A significant decrease in the melting temperature of the triplex was observed in both cases, being larger (−22.4°C) for the latter, demonstrating thus the high selectivity in the binding of sulphate modified PNAs. In parallel the same effect was investigated on hybrids formed by PNA and DNA oligomers by measuring their melting temperature. The triplexes (DNA)_2_DNA mis S and (PNA)_2_DNA mis S have a melting temperature respectively 15.4°C and 19.7°C lower than the perfectly matched hybrid. Furthermore, as the thermal stability of DNA hybrids depends on the ionic strength, unlike that of hybrids formed by standard PNA, [44] being increased at high ionic strength, we investigated the effect of the ionic strength on the stability of the PNA S hybrid. The melting temperature of (PNA S)_2_ DNA was found dependent on the ionic strength, being increased by about 4°C when the concentrations of NaCl and MgCl_2_ are doubled (Table 3).

**Table 3:** Melting temperature of the triplexes dissolved in 10 mM Na Phosphate buffer, 100 mM NaCl, 5 mM MgCl_2_, pH 7; * higher ionic strength: 10 mM Na Phosphate buffer, 200 mM NaCl, 10 mM MgCl_2_, pH 7.

triplex	(1)_2_5	(1)_2_6	(1)_2_7	(2)_2_5	(2)_2_7	(4)_2_5	(4)_2_7	(1)_2_5 *
Tm	46.6	29.6	24.2	52.2	32.5	42.2	26.8	50.8

### Cytotoxicity Assay

We performed a cytotoxicity assay on SKBR3 cells with increasing concentrations of PNA S. Results showed no significant difference on cell survival with respect to SKBR3 grown in the absence of the oligonucleotide analogues (data not shown). Thus, these data demonstrate that there is no intrinsic toxicity of the sulphate PNA on our cellular model.

### Cellular Uptake of FITC-oligomers

**Figure 7:**
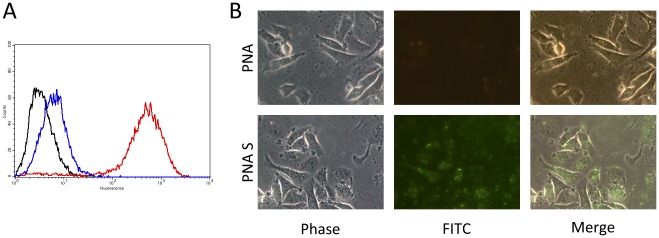
Uptake analysis of FITC-oligomers in SKBR3 cells. Cells are treated with 1 µM FITC-PNA S or FITC-PNA in the presence of lipofectamine and analysed by flow cytometric assay (**A**) and immunofluorescence analysis (**B**). In panel A, black curve represent not transfected cells, blue curve cells transfected with FITC-PNA, red curve cells treated with FITC-PNA S.

To evaluate the cellular uptake of PNA S and PNA in the presence of lipofectamine, SKBR3 cells were treated with FITC labelled molecules and analysed by flow cytometry and fluorescence microscopy. Fluorescence of cells treated with FITC-PNA S is markedly higher with respect to that of cells that incorporated PNA, on equal conditions as demonstrated and quantified by FACS analyses (Figure 7, Panel A) and confirmed by fluorescence microscopy experiments (Figure 7, panel B). These results clearly demonstrate the more efficient uptake of the PNA S in SKBR3 cells with respect to PNA mediated by lipofectamine.

### qPCR

**Figure 8:**
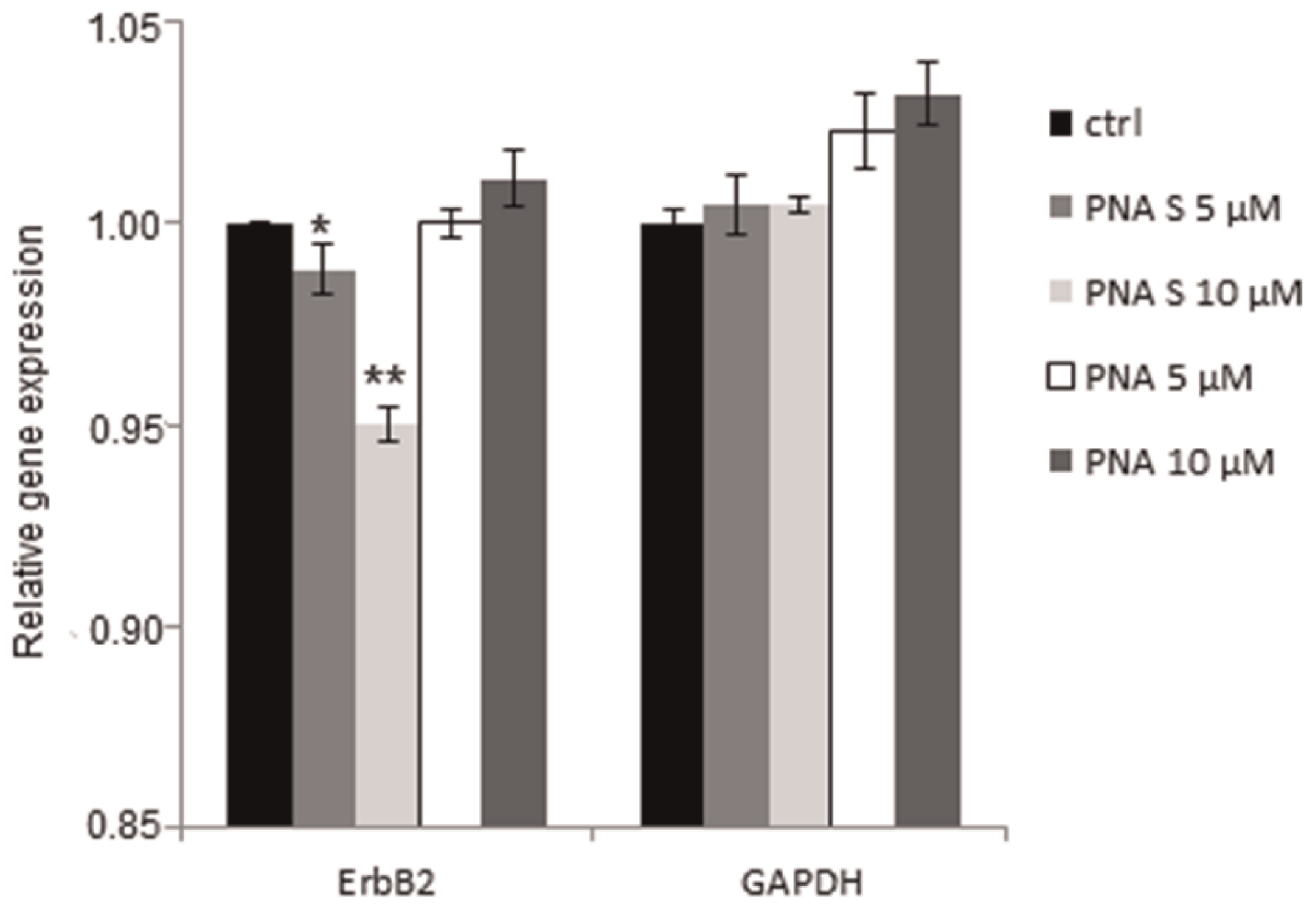
qPCR of ErbB2 and GAPDH genes. RNA was obtained after transfection of SKBR3 cells with PNA S or PNA at 5 and 10 µM concentration. The experiments were performed in triplicate and repeated at least 3 times. Data indicate the relative gene expression of cells treated with PNA S or PNA versus not treated cells, error bars represent the standard deviation of the mean. Statistical significance was carried out by means of the two tailed paired Student’s t test, *p = 0.02; **p = 0.0006.

A quantitative analysis of *ErbB2* expression was obtained by qPCR. In detail, SKBR3 cells were transfected for 48 h with different amounts of PNA S or PNA in the presence of lipofectamine and then total RNA was extracted from cells. Following the reverse transcription, performed using random primers and MMLV reverse-transcriptase, a qPCR reaction was carried out using specific primers for glyceraldehyde-3-phosphate dehydrogenase (GAPDH, a housekeeping gene, used as reference gene) and *ErbB2*. Data from this analysis showed that the PNA S was able to reproducibly down-regulate *ErbB2* expression at all concentrations tested. The expression of *ErbB2* in the presence of 10 µM PNA S was significantly lower than that at 5 µM. In contrast, treatment with PNA does not reduce ErbB2 gene expression (Figure 8).

### Flow Cytometric Analysis

**Figure 9:**
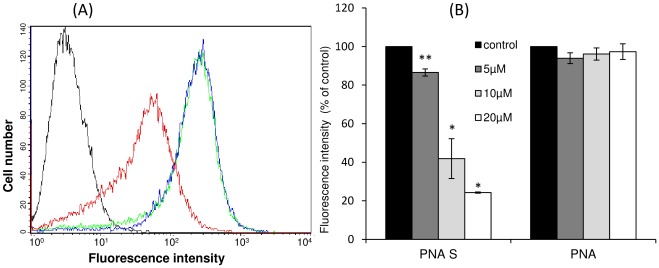
Flow cytometric analysis of ErbB2 expression on SKBR3 cells. Cells are treated with different amounts of PNA S and PNA in the presence of lipofectamine. (**A**) Single result representative of three similar experiments. Not transfected cells (black curve), not transfected and treated cells with an antiErbB2 antibody (green curve), transfected with 10 µM PNA S and treated with an antiErbB2 antibody (red curve) and transfected with 10 µM PNA and treated with an antiErbB2 antibody (blue curve). (**B**) Histograms were obtained from at least three independent experiments. Data are expressed as percentage of decrease versus control (cells not transfected and treated with an antiErbB2 antibody) ± SE. Statistical significance was carried out by means of the two tailed paired Student’s t test, **p<0.01, *p<0.05.

The ability of PNA S to recognize a specific DNA sequence was determined by analyzing its effect on the reduction of the amount of ErbB2 receptor on cell surface of SKBR3 cells overexpressing ErbB2 by flow cytometric assays. In particular, SKBR3 cells were transfected with PNA S in the presence of lipofectamine at different concentrations of the compound. The cells, harvested after 48 h, were incubated in the presence of Herceptin as primary antibody. The signal, indicating the binding of Herceptin to the receptor on SKBR3 cell surface, significantly decreases in the presence of increasing amount of PNA S. In contrast, there was no signal change when cells were transfected with PNA in the same conditions. This experiment suggests that the PNA S, unlike PNA, is able to inhibit the expression of the receptor in a dose-response manner (Figure 9).

## Discussion

In this paper a new PNA analogue is investigated: for the first time a sulphate group has been attached to the PNA backbone and the effects of the modification on the monomers conformation and oligomer hybridization properties have been analyzed. The position γ of the backbone was chosen based on data reported in the literature showing that the presence of substituents in the γ position does not hamper formation of duplex and triplex hybrids, and contributes to the oligomer structural organization. We employed NMR tools to determine the conformation of the monomers. Two rotamers were identified for each monomer, as reported for other γ modified PNAs. Analysis of the NOESY spectra recorded for the sulphate monomers and comparison with the spectra of hydroxymethyl modified monomers reveals a clear difference in the population of the rotamers, which suggests that the sulphate monomers, unlike the hydroxymethyl precursors, prefer the conformation in which the C-7 carbonyl group points toward the C-terminus. DQ-COSY experiments on the sulphate monomers, revealed that the two different rotamers existing for each monomer show two different conformations, respectively the right handed helix and the extended conformation. These data suggest that the introduction of a sulphate in the γ position of the backbone partially modifies the conformational preferences of γ modified hindered monomers. Interestingly, the conformation of rotamer A reproduces the situation observed in valine and isoleucine γ modified monomers. [26] The restricted rotation around C2–C3 forces the monomer in a specific conformation, corresponding to the one adopted in a right handed helix. On the other hand, rotamer B, assumes a more extended conformation, as demonstrated by the inversion in the pattern of coupling constants. Thus the sulphate side chain reproduces an intermediate situation between that observed in very hindered monomers as valine and isoleucine PNAs and in monomers with less hindered side chains as alanine and hydroxymethyl. The effect of the sulphate side chain on the secondary structure of the modified oligomer was investigated by CD, comparing the spectra for PNA S and PNA OH. It is reported in the literature that PNA oligomers containing γ hydroxymethyl monomers adopt the structure of a right handed helix. [19] The spectra recorded for PNA S and PNA OH show bands in the same position, with the bands in PNA S spectrum being far less intense (Figure 5). This result suggests that the PNA S oligomer has a lower content of secondary structure as compared to the PNA OH oligomer. This likely reflects the presence of a population of monomers which assumes an extended conformation, as assessed by NMR, and does not induce any secondary structure in the oligomer. Furthermore it is also reasonable to hypothesize that the electrostatic repulsions between the negative charges and the steric hindrance of sulphates in the backbone contribute to the helix unwinding in PNA S. The lack of a defined secondary structure in the single stranded PNA S does not affect its ability to form hybrids with complementary DNA. Analysis of the stoichiometry of the complexes by the continuous variation method reveals that the homopyrimidine PNA S forms triplexes with DNA, following the standard hybridization scheme (PNA 2:DNA). The CD signature of the PNA S triplex is superimposable upon that of a standard PNA triplex, suggesting that PNA S triplex assumes the secondary structure of a P-helix. Interestingly the PNA S triplex is thermally more stable than the DNA triplex. However a destabilization with respect to the standard PNA hybrid is observed, which can be explained considering the electrostatic repulsion between the negatively charged phosphate of DNA and sulphate of PNA S. The remarkable decrease in the melting temperature of PNA S hybrids containing a single mismatch in correspondence of the sulphate monomer indicates that it has the highest selectivity in the binding of DNA as compared to PNA and DNA. The hybridization properties displayed by PNA S urged us to explore its biological activity. PNA S is complementary to a tract of the *ErbB2* gene promoter and, therefore, its ability to work as an antigene may be investigated. To this end qPCR and FACS experiments were carried out to determine the amounts respectively of the ErbB2 gene expressed and the ErbB2 protein produced on the surface of SKBR3 cells.

qPCR and FACS analyses demonstrate that a treatment of SKBR3 cells with PNA S determines a significant decrease both in gene expression and in the amount of receptor on SKBR3 cell surface. These results suggest that PNA S binds to the *ErbB2* promoter, likely forming a triplex, and acts as an antigene.

Fluorescence experiments carried out on fluorescein labeled PNA and PNA S complexed to lipofectamine on SKB3 cells, reveal that cells intake PNA S to a great extent, whereas the PNA uptake seems to be poor.

Interestingly PNA S forms triplexes less stable than the standard PNA, but it exhibits a biological activity unlike PNA. One of the major challenges in the design of an antigene molecule is the optimization of its DNA affinity binding, which is achieved in several ways, for example conjugating to the PNA intercalating, chelating and alkylating agents, or using bis PNA. [37], [45], [46], [47], [48], [49], [50], [51] The results of the experiments carried out with PNA S highlight the importance of the specificity in the DNA binding, other than the affinity. Finally, in this case it can be hypothesized that the differences between PNA and PNA S are due to the different uptake of the molecules as well: the negatively charged PNA S reasonably binds strongly the cationic lipids used for the delivery, being able to reach the nucleus in a sufficient amount to determine the antigene effect, unlike PNA which, lacking of charge, is not efficiently delivered to the nucleus and therefore does not display any biological activity. Our hypothesis is consistent with data recently published in the literature [9] and is supported by the results of fluorescence experiments; other assays, as for example the Chromatin Association Assay might be carried out to demonstrate that PNA S bind to DNA *in vivo*. [52] Data obtained so far underline how critical is the efficiency in the uptake to determine the biological activity of PNA analogues. In conclusion PNA S is the first example reported so far of antigene γ PNA bearing a negative charge. The introduction of the sulphate group in the γ position of the backbone positively influences the binding properties and the biological activity of the oligomer. PNA S shows a strong binding selectivity toward DNA and a potent antigene activity. The lack of toxicity encourage us to further investigate the application of PNA S in strategies aimed at interfering in gene expression, increasing the length and consequently the specificity of PNA oligomers in order to target unique sites in DNA/RNA binding sites.

## Methods

### Synthesis of the Monomers

Sulphate monomers were obtained after derivatization of the γ hydroxymethyl monomer, synthesized following procedures described in the literature. [19] The protocol to install the sulphate is here described for the a^S^ monomer, but applies to all other monomers as well.

Fmoc Ser(OH)-A(Bz)-OH (Fmoc-a^OH^-OH) (143 mg, 0.22 mmol) is dissolved in 1.2 mL of DMF and stirred under argon 10 minutes. DMF·SO_3_ (134.8 mg, 0.88 mmol) is added and the reaction is carried out for 2 hours under argon at r.t. The solvent is evaporated. Separately a solution of NaHCO_3_ sat.(2.86 mL) and tetrabutylammonium bisulfate (154.4 mg) is prepared and cooled at 0°C; this solution is added to the reaction mixture and reacted under stirring for 5 minutes. The pH of the solution is then adjusted to 5 with a 10% solution of citric acid. The crude is extracted 4 times with CHCl_3_ and the organic layer is dried under vacuum. Yield: 95%.

### NMR Spectroscopy

The 2D spectra were acquired using the TPPI (time-proportional phase-incrementation) method to obtain complex data points in the t_1_ dimension. A standard set of 2D experiments DQF-COSY, TOCSY and NOESY were acquired at 25°C. [53], [54], [55] The TOCSY experiments were recorded using a MLEV17 mixing scheme of 70 ms with 9 kHz spin-lock field strength; the NOESY spectra were carried out with a mixing time of 150 ms. Chemical shifts were referenced to external TMS (tetramethylsylane) (δ = 0 ppm). For data processing and spectral analysis the programs VNMR 6.1B and CARA were used. [56] All samples were purified by HPLC before analysis.

### Determination of the Stoichiometry of the Complex by Job Plot

Concentration of the single strands was determined by UV at 260 nm, using for PNA S and PNA ε = 64.5 Lmol^−1^cm^−1^ and for DNA ε = 101.6 Lmol^−1^cm^−1^. 3 µM solutions of PNA S, PNA and DNAc in phosphate buffer 10 mM, 100 mM NaCl, pH = 7 were prepared. Solutions at different molar fraction of PNA S (or PNA) and DNAc were prepared, keeping constant the (PNA S or PNA + DNA) total concentrations. Samples were annealed, warming to 90°C and slowly cooling to 4°C. UV spectra were recorded on a Jasco spectrophotometer at 25°C.

### CD Analyses

CD spectra were recorded at 5°C in the 320–200 nm range and are the results of 6 scans. Single strands and triplexes were dissolved in phosphate buffer 10 mM, 100 mM NaCl, 5 mM MgCl_2_ pH = 7. Triplexes were annealed by warming up at 90°C and slowly cooling to 4°C.

Thermic denaturation experiments were carried out using 3 µM triplexes at a 0.5°C/min scan speed, recording at 225 nm for PNA S and PNA and at 266 nm for DNA triplexes.

### Cell Culture

SKBR3 cells (ATCC, U.S.) were grown in DMEM supplemented with 10% fetal bovin serum (FBS), 1% glutamine, 100 U/mL penicillin and 100 µg/mL streptomycin (Invitrogen, U.S.), at 37°C and 5% CO_2_.

### Citotoxicity Assay

For the evaluation of the cytotoxic effect of compounds, the exponentially growing cells were seeded at a density of 5.0×10^3^/well in 96-well flat bottom tissue culture microplates, and incubated with 1, 5 and 10 µM of PNA S at 37°C for 72 hours. Cell number was evaluated with crystal violet, which correlates optical density with cell number, according to the procedure described by Gilles et al. [57].

### Cellular Uptake by FACS and Fluorescence Microscopy

7.5×10^4^ cells/well were plated in 24-well plates with or without cover glasses, allowed to adhere for 24 hours, and then transfected using Lipofectamine LTX (Invitrogen, CA, U.S.) according to the manufacturer’s instructions, with FITC-PNA S or FITC-PNA. After 24 hours, cells were detached, then washed with PBS 1X, 0.1% BSA and analyzed by flow cytometer, equipped with a 488 nm argon laser (FACScalibur Becton Dickinson, U.S.). For each sample 10.000 events were acquired and analyzed using Cell Quest software. At the same time, cover glass adherent cells were fixed with 4% formaldehyde and analyzed by Axiovert 200 Zeiss microscopy fluorescence.

### RT-PCR and qPCR

Total RNA was extracted from lysates of transfected cells by using Tri-reagent™ (Sigma Aldrich, U.S.) according to the manufacturer’s instructions. Reverse transcription was performed using 0.5 µg of total RNA, 200 U of MMLV (Finnzymes, Finland) and 250 ng of random primers (Fermentas, Germany). Reaction temperature was set at 42°C for 1 hour. The reaction buffer was: 50 mM Tris HCl pH 8.3; 75 mM KCl; 3 mM MgCl_2_; 5 mM DTT. After reverse transcription, qPCR assay was carried out using the following primers, GAPDH: forward primer, 5′-ATGGGGAAGGTGAAGGTC-3′, reverse primer 5′-GTCATGGATGACCTTGGC-3′; ErbB2: forward primer, 5′-GGGAAGAATGGGGTCGTCAAA-3′, reverse primer 5′-CTCCTCCCTGGGGTGTCAAGT-3′ (purchased from Sigma-Genosys Ltd). The amplification reactions were run at least in triplicate. The reactions contained 50 ng of cDNA, 3 µl of primers (10 µM each), and 10 µl of SYBR Premix Ex Taq II (Takara, Japan), in a final volume of 25 µl. The qPCR protocol was as follows: 2 minutes at 95°C followed by 45 cycles of 1 minute at 95°C, 1 minute at 60°C and 1 minute at 72°C and the experiment was performed in Rotor-gene Q (Qiagen).

#### SKBR3 binding assay by fluorescence activated cell sorting (FACS)

7.5×10^4^ cells/well were plated in 24-well plates, allowed to adhere for 24 hours, and then transfected using Lipofectamine LTX with PNA S or PNA. After 48 hours, cells were incubated with 10 nM Herceptin (kindly provided by Dr. S. De Luca, IBB, CNR Italy), then washed with PBS 1X, 0.1% BSA and incubated with a 1∶1000 dilution of primary antibody (mouse anti-human, clone HP6017, SIGMA, U.S.). Secondary antibody (goat anti-mouse FITC-conjugate, Santa Cruz, CA, U.S.) was used at 1∶200 dilution. Subsequently cells were detached, resuspended in PBS 1X, 0.1% BSA and analyzed by flow cytometer. For each sample 20.000 events were acquired and analyzed using Cell Quest software.

## Supporting Information

Figure S1 **HPLC profile for the pure PNA S.**
(TIF)

Figure S2 **HPLC, mass spec and UV profile of FITC-PNA.**
(TIF)

Figure S3 **HPLC and UV profile of FITC-PNA S.**
(TIF)

Supporting Information S1 **Synthesis of the monomers.**
(DOCX)

Supporting Information S2 **Solid phase synthesis of PNA, PNA S and PNA OH.**
(DOCX)

Supporting Information S3 **Solid phase synthesis of FITC labeled PNA and PNA S.**
(DOCX)
