# Supplementary figures and images for: γ sulphate PNA (PNA S): Highly Selective DNA Binding Molecule Showing Promising Antigene Activity
Source: PLoS One. 2012 May 7;7(5):e35774. doi: 10.1371/journal.pone.0035774 (PMC3346730; doi:10.1371/journal.pone.0035774)

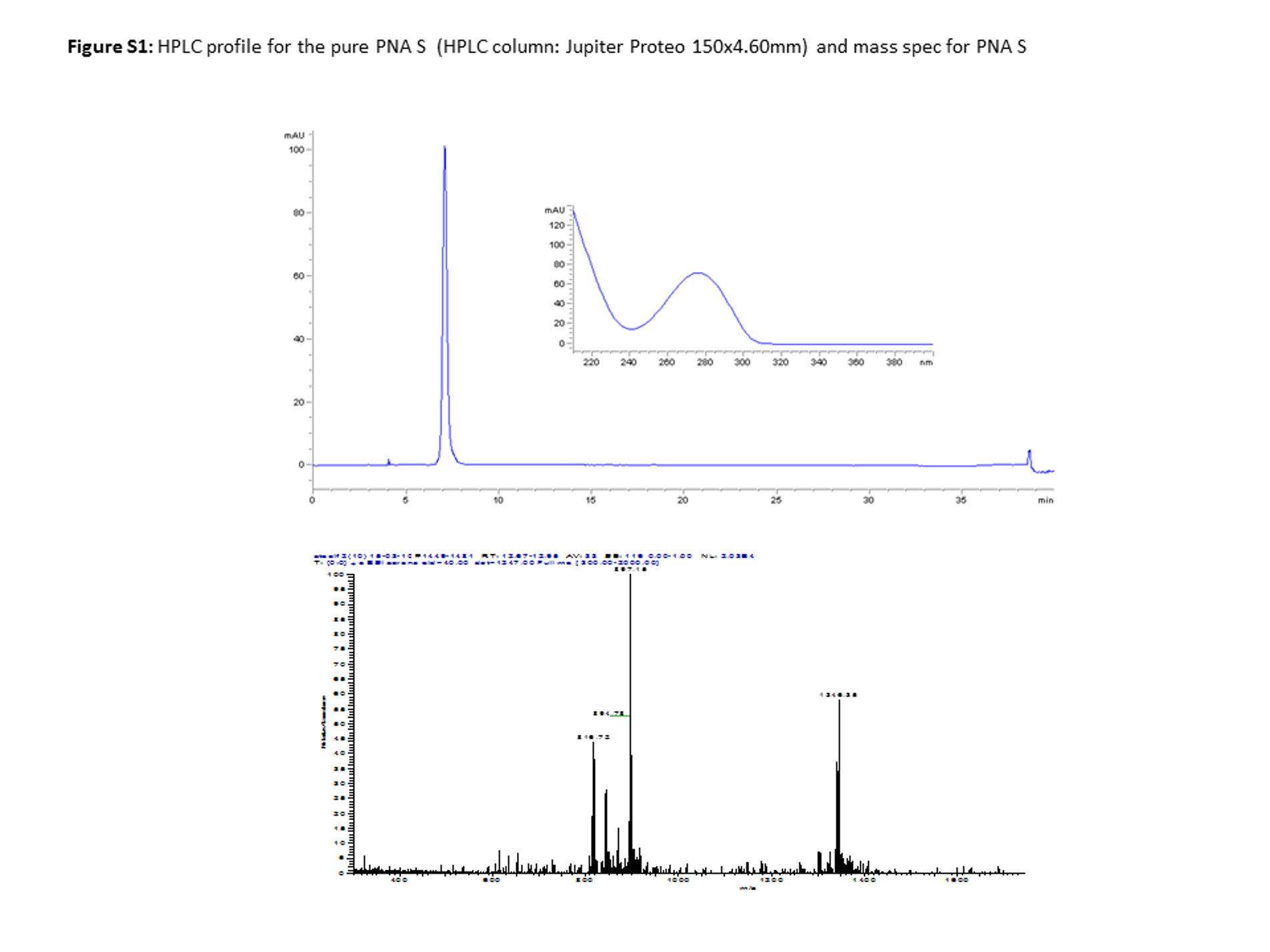

Supplement: Figure S1 — HPLC profile for the pure PNA S. (TIF) [file pone.0035774.s001.tif]

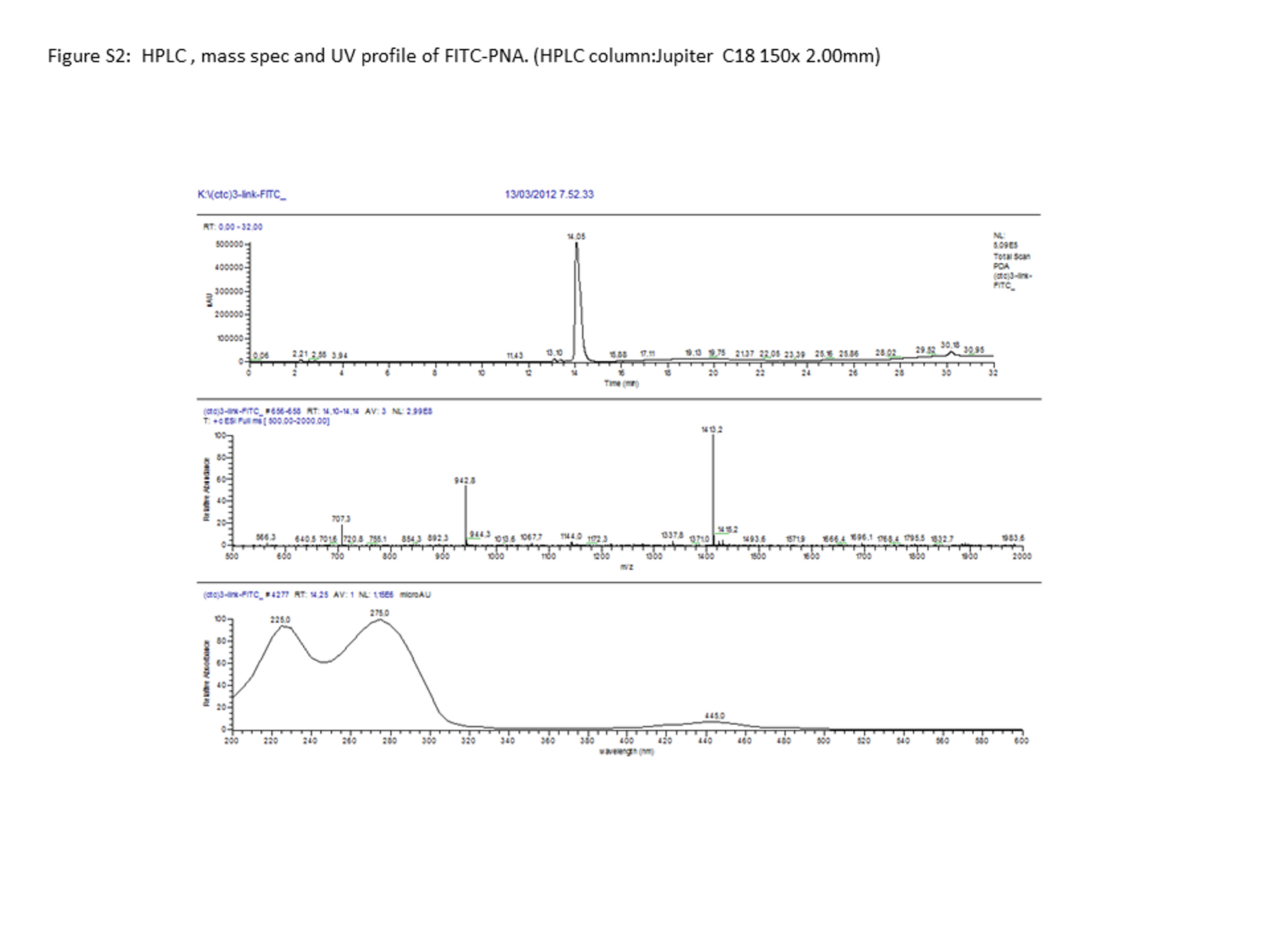

Supplement: Figure S2 — HPLC, mass spec and UV profile of FITC-PNA. (TIF) [file pone.0035774.s002.tif]

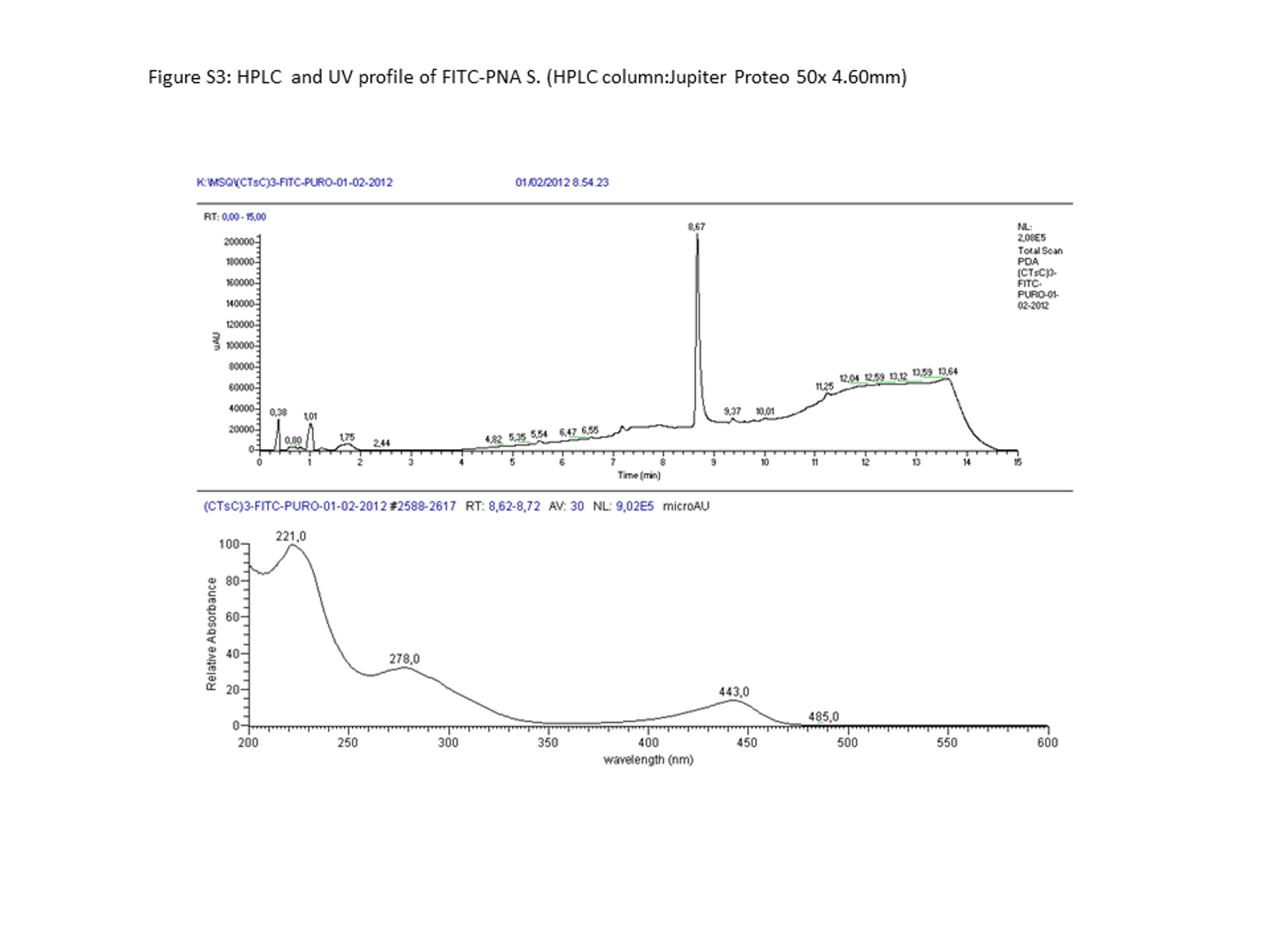

Supplement: Figure S3 — HPLC and UV profile of FITC-PNA S. (TIF) [file pone.0035774.s003.tif]
