# Supplementary material for: γ sulphate PNA (PNA S): Highly Selective DNA Binding Molecule Showing Promising Antigene Activity
Source: PLoS One. 2012 May 7;7(5):e35774. doi: 10.1371/journal.pone.0035774 (PMC3346730; doi:10.1371/journal.pone.0035774)
Supplement: Supporting Information S1 — Synthesis of the monomers. (DOCX) [file pone.0035774.s004.docx]

**S1 Synthesis of the monomers**

Reagents for the synthesis and solvents were purchased at Sigma-Aldrich. Activators (HBTU and HOBt) and amino acids were purchased at Novabiochem. DIPEA, DMF, DCM were from Romil and piperidine from Biosolve. Fmoc-PAL-PEG-PS resin was from Applied Biosystems. Fmoc-T-OH and Fmoc-C(Bhoc)-OH PNA monomers were purchased at Link Technologies. Analytical RP-HPLC runs were carried out on a HP Agilent Series 1200 apparatus using a Phenomenex Jupiter 5μ C18 300Å, 250x4.6 mm column with a flow rate of 1.0 mL min^-1^. LCMS analyses were run on a Thermo Finnigan instrument equipped with a MSQ ES source using a Phenomenex Jupiter 5μ C18 300Å , 150x4.6 mm column with a flow rate of 0.8 mL min^-1^.

Preparative RP-HPLC was carried out on a Shimadzu 8A apparatus equipped with an UV Shimadzu detector using a Phenomenex Jupiter 10μ Proteo 90Å, 250x10 mm column.

Circular dichroism spectra were recorded on a Jasco J-810 spectropolarimeter equipped with a Peltier thermal controller unity using a 1 cm quartz cell.

Monodimensional ^1^H- and ^13^C-NMR spectra were recorded on a Varian Innova instrument (600 MHz) at room temperature. All chemical shifts are expressed in ppm with respect to the signals of the residual protonated solvents (CDCl_3_ or DMSO-d_6_). Bidimensional NMR experiments were performed on a Varian *^UNITY^INOVA* 500 spectrometer, equipped with a 5-mm triple resonance probe and triple-axis pulsed-field gradients.

**S1 Synthesis of the monomers**

Scheme 1: Synthesis of the backbone

Scheme 2: Protected bases

Scheme 3: assembly of the monomers

**Fmoc-Ser(tBu)-N(OCH_3_)CH_3_ (2a)**

Fmoc-Ser(tBu)-OH (**1**) (1.5 g, 3.9 mmol) was dissolved in dry CH_2_Cl_2_ (52 mL). N,O-(dimethyl)-hydroxylamine chloridrate (0.507 mg, 5.2 mmol) and DMAP (47.8 mg, 0.39 mmol) were added and the mixture was cooled at 0 °C. Triethylamine (933 μL, 0.678 g) and EDC (0.9 g; 4.69 mmol) were added. The reaction was stirred 1 hour at 0 °C and 19 hours at room temperature. The crude was extracted with HCl 1N first, NaHCO_3_ aq. 10% and finally with H_2_O; the organic layer was treated with Na_2_SO_4_ an. , filtered and concentrated. The product was isolated after purification by silica gel chromatography, eluted with ethylic ether /petroleum ether 1/1 v/v.

Yield: 90% (1.66 g)

Mass analysis: m/z (ESI), [M+H]^+^ calculated: 426.4 ; found: 426.5

^1^H NMR(CDCl_3_): 1.16 ( 9 H, s, t-butyl-CH_3_)_,_ 3.24 (3H, s, -N-CH_3_-), 3.60-3.65 (2H, m, -CH-CH_2_-O-), 3.77 (3H, s, -O-CH_3_), 4.23 (1H, t, Fmoc-CH-CH_2_-), 4.34 (2H, d, Fmoc- CH_2_-O-), 4.87 (1H, t, -NH-CH-CH_2_- O-), 7.21-7.74 (8H, m, Fmoc)

^13^C NMR (CDCl_3_): 27.34 (t-butyl-CH_3_-), 47.15 (-N-CH_3_), 51.80 (Fmoc-CH), 61.48 (-NH-CH-C-), 62.04 (-O-CH_3_-), 67.10 (-CH_2_-O-Fmoc), 73.58 (t-butyl-C-), 125.23, 125.21, 127.05, 127.66, 141.27, 143.87 (Fmoc), 144.00 (-CH_2_-COO-N), 156.0 (-O-COO-NH).

**Fmoc-Ser(tBu)-OCH_3_  (3)**

Fmoc-Ser(tBu)N(OCH_3_)CH_3_ **(2a)** (0.78 g; 1.8 mmol) dissolved in dry diethyl ether (11 mL) was cooled at 0 °C under argon. Lithium aluminum hydride (3.5 mL; 3.5mmol) was added dropwise and the reaction was stirred at 0 °C, 1 hour. The reaction was quenched with a solution of KHSO_4_ (0.2 M); the crude was extracted with H_2_O. The organic layer containing the aldehyde Fmoc-Ser(tBu)-H (2b) was treated with Na_2_SO_4_ an. , filtered and concentrated.

Fmoc-Ser(tBu)-H(**2b**) (0.58 g; 1.5 mmol) dissolved in anhydrous CH_3_OH (16 mL) was cooled at 0 °C under nitrogen. In a separate flask glycine methylester chlorhydrate (0.4 g; 3.1 mmol) was dissolved in CH_3_OH (2 mL) and DIPEA (500 μL). The two solutions were mixed and the reaction was stirred at 4 °C, 4 hours . Acetic acid (290 μL) and sodium cianoborhydride (152 mg; 2.41 mmol) were then added and the mixture was stirred 30 minutes. The reaction was quenched with a solution of NaHCO_3_ 10%; the crude was extracted with ethyl acetate. The organic layer was treated with Na_2_SO_4_ an. , filtered and concentrated. Product **3** was purified by silica gel chromatography eluted in ethyl acetate/hexane7/3 v/v.

Yield: 60 % (0.41g)

Mass analysis: m/z (ESI), [M+H]^+^ calculated : 443.0 ; found :443.1

^1^H NMR (CDCl_3_): 1.17 ( 9H, s, t-butyl-CH_3_), 2.11 (1H, s, -CH_2_- NH-CH_2_-), 2.74 (2H, dd, -CH-CH_2_-N-), 3.44 (2H, bs, NH-CH_2_-COO), 3.47 (2H, br s, -CH-CH_2_-O-), 3.70 (3H, s, -O-CH_3_), 3.88 (1H, br s, -NH-CH-CH_2_-), 4.23 (1H, m, Fmoc- CH-CH_2_), 4.40 (2H, m, Fmoc-CH_2_-O), 7.28-7.76 (8H, m, Fmoc).

^13^C NMR(CDCl_3_): 27.62 (t-butyl-CH_3_-), 47.42 (Fmoc-CH-CH_2_), 50.92 (-CH-CH_2_-N-), 51.06 ( -NH-CH_2_-CO), 52.15 (-O-CH_3_), 62.25 (NH-CH-CH_2_-), 67.10 (Fmoc-CH_2_-O-), 73.36 (CH-CH_2_-O-), 125.33, 127.25, 127.85 (Fmoc), 144.10, 144.24 (Fmoc), 156.85 (-O-CO-N-) , 173.75 (COOCH_3_)

**Fmoc-Ser(tBu)-A(Bz)-OCH_3_ (8B)**

0.17 g of Fmoc-Ser(tBu)-OCH_3_ **(3)**(0.37 mmol) are dissolved in 3.7 mL of anhydrous DMF; A(Bz)-CH_2_COOH (224 mg, 0.75 mmol)(prepared following the protocol reported by Finn, PJ et al. *Nucleic Acids Research* **(**1996)) EDC (144.3 mg, 0.75 mmol ) and DMAP (4.6 mg, 0.04 mmol) are added. After 12 hours stirring the solvent is evaporated; the residue is dissolved in CHCl_3_ and washed with NaHCO_3 aq._ The organic layer is treated with Na_2_SO_4_ and the solvent evaporated. The product is purified by silica gel chromatography eluted in ethyl acetate/hexane 9/1 v/v.

Yield: 85% (225 mg)

Mass analysis: m/z (ESI), [M+H]^+^ calculated 698.0; found 698.2

^1^H NMR(CDCl_3_): 1.26 (9H, s, t-butyl-CH_3_), 3.41–3.70 ( 4H, m, -CH–CH_2_–O-N-CH-CH_2_), 3.72 (3H, s, OCH_3_), 3.82 (2H, s, CO-CH_2_-adenine), 3.97-4.20 (3H, m, NH-CH-CH_2_, NH-CH_2_-COOCH_3_), 4.28 (1H, t, CH-Fmoc), 4.40-4.41 (2H, d, CH_2_-Fmoc), 7.28-7.75 (8H, m, Fmoc and 5H, m, Bz), 8.12 (1H, s, H(2)-adenine), 8.68 (1H, s, H, (8)-adenine)

^13^C NMR (CDCl_3_): 27.90 (t-butyl-CH_3_), 44.22 (N-CH_2_-COO), 47.71 (Fmoc-CH-), 49.26 (CH-CH_2_-N), 49.98 (-NH-CH-CH_2_), 52.84 (OCH_3_), 60.55 (-CO-CH_2_-adenine), 67.20 (-Fmoc -CH_2_-), 67.43 (CH-CH_2_-O), 74.58 (t-butyl-C), 120.52 (C(5)-adenine), 125.32, 127.55, 128.31, 129.34, 133.19, 134.30, 141.81, 144.14 ( Fmoc), 144.64 (C(8)-adenine), 149.87 (C(4)-adenine), 153.17 (C(2)-adenine), 155.00 (CO-Fmoc), 165.00 (CO-Bz), 169.83 (-CO-CH_2_-adenine), 173.06 (COOCH_3_).

**Fmoc Ser(tBu)-A(Bz)-OH (10 B)**

224 mg of Fmoc-Ser(tBu)-A(Bz)-OCH_3_ (**8B**)(0.32 mmol) are dissolved in dioxane (4 mL) and treated with NaOH 2M ( 802 μL, 1.6mol). The mixture is stirred at r.t. and 0 °C, 2 hours; HCl 1N is added until the pH of the solution is 5. The product is extracted with ethyl acetate and dried. The resulting NH_2_-Ser(tBu)-A(Bz)OH (156 mg, 0.32 mmol) is dissolved in 2.5 mL of H_2_O/CH_3_CN 1/1, FmocONSu (216.6 mg, 0.64 mmol) and NaHCO_3_ are added (54 mg, 0.64 mmol). The reaction is carried out at pH=8, 2 hours. The reaction is quenched with HCl 2M up to neutralization. The crude is washed with CHCl_3_, the organic layer is dried. The product is purified by by silica gel chromatography eluted in ethyl acetate/methanol 9/1 v/v.

Yield: 63% (143mg)

Mass analysis: m/z (ESI), [M+H]+ calculated: 707.0; found: 707.2

^1^H NMR (DMSO-d_6_): 1.21 (9H, s, t-butyl-CH_3_), 3.14-4.14 (7H, m, -CH-CH_2_-O, NH-CH-CH_2_, NH-CH-CH_2_, CO-CH_2_-adenine), 4.19-4.41 (5H, m, CH-Fmoc, CH_2_-Fmoc, NH-CH_2_-COOH), 7.28-8.05 (8H, m, Fmoc and 5H, m, Bz), 8.30 (1H, s, H(2) adenine), 8.62 (1H, s, H(8) adenine).

^13^C NMR (DMSO-d_6_): 31.02 (t-butyl-CH_3_), 47.90 (Fmoc-CH), 50.73 (COCH_2_-adenine), 52.15 (NH-CH_2_-COOH), 53.36 (CH-CH_2_-N), 54.72 (NH-CH-CH_2_), 69.00 (Fmoc-CH_2_-), 69.47 (CH-CH_2_-O), 76.82 (t-butyl-C), 124.09 (C(5)-adenine), 129.17, 130.99, 131.59, 132.42, 136.36 , 144.71 ( Fmoc, Bz), 137.41 (C(8)-adenine), 144.71 (C(4)-adenine), 149.50 (C(6)-adenine), 153.90 (C(2)-adenine), 156.73 (COO-Fmoc), 169.64 (CO-Bz), 170.80 (CO-CH_2_-adenine), 174.08 (COOH)

**Fmoc Ser(OH)-A(Bz)-OH (11B)**

Fmoc Ser(tBu)-A(Bz)-OH (**10B**) (143 mg) is suspended in TFA/DCM 1/1 (3 mL); the solution is stirred 2 hours. The solvent is evaporated and the crude is lyophilized. Yield: 100% (131.5 mg)

Mass analysis: m/z (ESI), [M+H]^+^ calculated :650.0; found: 650.3

^1^H NMR: (DMSO-d_6_): 3.19 - 3.36 (2H, m, CH-CH_2_-N, rotamer B), 3.57-3.49 (2H, m, CH-CH_2_-N, rotamer A), 3.60 (2H, m, CH-CH_2_-O, rotamer B), 3.70 (1H, m, NH-CH-CH_2_ rotamer B), 3.72 (2H, m, CH-CH_2_-O, rotamer A), 3.88 (1H, m, NH-CH-CH_2_ rotamer A), 3.96-4.08 (2H, d, N-CH_2_-COOH), 4.24-4.34 (3H, m, CH-Fmoc, CH_2_-Fmoc), 5.20-5.26 (2H, d, CO-CH_2_-adenine, rotamer B), 5.42-5.50 (2H, d, CO-CH_2_-adenine, rotamer A), 7.12 (1H, d, NH-CH-CH_2_ rotamer B), 7.25-8.10 (8H, m, Fmoc and 5H, m, Bz), 8.48 (1H, s, H(2)adenine), 8.68 (1H, s, H(8)adenine). 7.41 (1H, d, NH-CH-CH_2_ rotamer A),

^13^C NMR (DMSO-d_6_): 47.70 (CH-Fmoc), 50.00 (CO-CH_2_-adenine), 52.05 (NH-CH_2_-CO), 53.20 (CH-CH_2_-N), 54.78 (NH-CH-CH_2_), 66.53 (CH-CH_2_-O), 69.40 (CH_2_-Fmoc), 121.08 (C(5)-adenine), 132.42, 129.44, 128.59, 128.00, 126.16 (Fmoc, Bz), 134.32 (C(8)-adenine), 144.79, 141.69 (Fmoc), 150.71 (C(4)-adenine), 152.41 (C(6)-adenine), 153.62 (C(2)-adenine), 156.83 (CO-Fmoc), 166.76 (CO-Bz), 167.71 (CO-CH_2_-adenine), 171.64 (COOH).

**Fmoc Ser(OSO_3_)-A(Bz)-OH (Fmoc-a^S^-OH) (12 B)**

Fmoc Ser(OH)-A(Bz)-OH (**11B**) (143 mg, 0.22 mmol) is dissolved in 1.2mL of DMF and stirred under argon 10 minutes. DMF·SO_3_ (134.8 mg, 0.88 mmol) is added and the reaction is carried out 2 hours under argon at r.t.. The solvent is evaporated. Separately a solution of NaHCO_3_ sat.(2.86 mL) and tetrabutylammonium bisulfate (154.4 mg) is prepared and cooled at 0 °C; this solution is added to the reaction mixture and and reacted under stirring 5 minutes. The pH of the solution is then adjusted to 5 with a 10% solution of citric acid. The crude is extracted 4 times with CHCl_3_, the organic layer is dried under vacuum.

Yield: 95%

Mass analysis: m/z (ESI), [M+H]^+^ calculated: 729.3; found: 729.4

**^1^H NMR** (DMSO-d_6_): 3.28 - 3.49 (2H, m, CH-CH_2_-N, rotamer B), 3.50-3.74 (2H, m, CH-CH_2_-N, rotamer A), 3.73 (2H, m, CH-CH_2_-O, rotamer B), 3.86 (1H, m, NH-CH-CH_2_ rotamer B), 3.90 (2H, m, CH-CH_2_-O, rotamer A), 3.92-4.04 (2H, d, N-CH_2_-COOH), 4.08 (1H, m, NH-CH-CH_2_ rotamer A), 4.24-4.34 (3H, m, CH-Fmoc, CH_2_-Fmoc), 5.11 (2H, sd, CO-CH_2_-adenine, rotamer B), 5.50-5.58 (2H, d, CO-CH_2_-adenine, rotamer A), 7.28-8.10 (8H, m, Fmoc and 5H,m, Bz), 7.35 (1H, d, NH-CH-CH_2_ rotamer B),7.62 (1H, d, NH-CH-CH_2_ rotamer A), 8.46 (1H, bs, H(2)adenine), 8.66 (1H, bs, H(8)adenine)

All other monomers are prepared following the same procedure described for the adenine monomer. Bases with the methylene carbonyl linker were prepared according to procedures described in the literature. ( Aldrian-Herrada, G. et al., 1998; Finn, P.J. et al. Nucleic Acids Research, 1996; Timar, Z. et al 2000)

**Fmoc-Ser(tBu)-T-OCH_3_ (8A)**

Yield: 89% (185.2 mg)

Mass analysis: m/z (ESI), [M+H]^+^ calculated 609 ; found 609.2

^1^H NMR (CDCl_3_): 1.21 (9H, s, t-butyl-CH_3_), 1.67-1.68 (3H, s, CH_3_-thymine), 3.42-3.90 (4H, m, -CH-CH_2_-O-), 3.72 (3H, s, O-CH_3_), 3.79 (2H, dd, N-CH_2_-COOCH_3_), 4.19-4.23 (1H, m, -NH-CH-CH_2_), 4.29 (1H, t, Fmoc-CH-CH_2_-), 4.41 (2H, d, Fmoc-CH_2_-O-), 4.54-4.81 (2H, s, CO-CH_2_- thymine), 6.96-6.98 (1H, s, H(6)- thymine), 7.29-7.77 (8H, m, Fmoc), 8.02 (1H, s, NH- thymine), 8.24 (1H, s, Fmoc-NH)

^13^C NMR (CDCl_3_): 12.33 (CH_3_- thymine), 27.50 (t-butil-CH_3_), 47.27 (N-CH_2_-CO), 47.56 (Fmoc-CH-CH_2_), 48.87 (CH-CH_2_-N), 49.60 (NH-CH-CH_2_-), 49.98 (-CO-CH_2_- thymine), 52.35 (-O-CH_3_), 66.74 (Fmoc-CH_2_-O), 66.96 (-CH-CH_2_-O-), 73.94 (t-butyl-C), 110.5 (C(5)- thymine), 125.00, 127.09, 127.80, 140.92, 141.18, 143.76 (Fmoc), 141.18 (C(6)- thymine), 150.75 (C(2)- thymine), 163.83 (C(4)- thymine), 167.75 (-CO-CH_2_- thymine), 169.51 (COOCH_3_)

**Fmoc Ser(tBu)-T-OH (10 A)**

Yield: 63% (223 mg)

Mass analysis: m/z (ESI), [M+H]^+^ calculated: 595.0; found: 595.2

^1^H NMR (DMSO-d_6_): 1.21 (9H, s, t-butyl-CH_3_), 1.67-1.68 (3H, s, CH_3_- thymine), 3.45-3.80 (4H, m,-CH-CH_2_-O, NH-CH-CH_2_), 3.82-3.98 (2H, s, NH-CH_2_-COOH), 4.21-4.45 (5H, m, CH-Fmoc, CH_2_-Fmoc, CO-CH_2_- thymine), 7.21 (1H, s, H(6)- thymine), 7.29-7.72 (8H, m, Fmoc)

^13^C NMR (CDCl_3_): 11.48 (CH_3_- thymine), 26.75 (t-butyl-CH_3_), 46.27 (N-CH_2_-COOH), 47.24 (CH-Fmoc), 48.09 (CH-CH_2_-N), 49.67 (NH-CH-CH_2_-), 50.21 (CO-CH_2_- thymine), 65.00 (Fmoc-CH_2_-O), 71.98 (CH-CH_2_-O), 75.73 (t-butyl-C), 107.56 (C(5)- thymine), 124.77, 126.59, 127.20, 141.62, 143.32 (Fmoc), 140.23 (C(6)- thymine), 150.54 (C(2)- thymine), 163.93 (C(4)- thymine), 167.75 (-CO-CH_2_- thymine), 170.54 (COOH)

**Fmoc Ser(OH)-T-OH (11 A)**

Yield: 86% (174mg)

Mass analysis: m/z (ESI), [M+H]^+^ calculated: 538.0 ; found: 538.2

^1^H NMR (DMSO-d_6_): 1.67 (3H, s, CH_3_-thymine), 3.13 -3.34 (2H, m, CH-CH_2_-N, rotamer B), 3.33 -3.49 (2H, m, CH-CH_2_-N, rotamer A), 3.56 (2H, m, CH-CH_2_-O, rotamer B), 3.57 (2H, m, CH-CH_2_-O, rotamer A), 3.67 (1H, m, NH-CH-CH_2_ rotamer B), 3.78 (1H, m, NH-CH-CH_2_ rotamer A), 3.92-3.99 (2H, d, N-CH_2_-COOH), 4.21-4.40 (3H, m, CH-Fmoc, CH_2_-Fmoc) , 4.65-4.73 (2H, d, CO-CH_2_-thymine), 7.10 (1H, d, NH-CH-CH_2_ rotamer B),7.26 (1H, bs, H(6)-thymine), 7.29-7.90 (8H, m, Fmoc), 7.31 (1H, d, NH-CH-CH_2_ rotamer A)

^13^C NMR (DMSO-d_6_): 15.91 (CH_3_-C5), 50.77 (CH-Fmoc), 51.79 (CO-CH_2_-thymine), 53.24 (NH-CH_2_-COOH), 55.55 (NH-CH-CH_2_), 55.89 (CH-CH_2_-N), 66.77 (CH_2_-Fmoc), 69.22 (CH-CH_2_-O-), 112.13 (C(5)- thymine), 124.15, 129.26, 131.10, 131.67 (Fmoc), 144.76, 147.88 (C_q_ Fmoc), 146.07 (C(6)- thymine), 155.01 (C(2)- thymine), 159.98 (COO-Fmoc), 168.43 (C(4)- thymine), 171.55 (CO-CH_2_- thymine), 174.28 (COOH)

**Fmoc Ser(OSO_3_)-T-OH (Fmoc-t^S^-OH) (12 A)**

Mass analysis: m/z (ESI), [M+H]^+^ calculated: 617.0 ; found: 618.0

**^1^H NMR** (DMSO-d_6_): 1.69 (3H, s, CH_3_-thymine), 3.32-3.47 (2H, m, CH-CH_2_-N, rotamer B), 3.40 - 3.58 (2H, m, CH-CH_2_-N, rotamer A), 3.55 (2H, m, CH-CH_2_-O, rotamer B), 3.77 (1H, m, NH-CH-CH_2_ rotamer B), 3.94-3.96 (2H, d, N-CH_2_-COOH), 3.97 (2H, m, CH-CH_2_-O, rotamer A), 4.08 (1H, m, NH-CH-CH_2_ rotamer A), 4.18-4.32 (3H, m, CH-Fmoc, CH_2_-Fmoc) , 4.45 (2H, d, CO-CH_2_-thymine, rotamer B) , 4.65-4.73 (2H, d, CO-CH_2_-thymine, rotamer A), 7.26-7.90 (8H, m, Fmoc), 7.31 (1H, bs, H(6)-thymine), 7.33 (1H, d, NH-CH-CH_2_ rotamer B), 7.52 (1H, d, NH-CH-CH_2_ rotamer A).

**Fmoc Ser(tBu)-C(Bz)-OCH_3_ (8C)**

Mass analysis: m/z (ESI), [M+H]^+^ calculated: 698.0 ; found: 698.2

^1^H NMR (CDCl_3_): 1.22 (9H, s, t-butyl-CH_3_), 3.30-3.40 (2H, m, CH-CH_2_-O), 3.45-3.54 (2H, m, CH-CH_2_-O), 3.71 (3H, s, OCH_3_), 3.78 (2H, s, CO-CH_2_-cytosine), 3.95-4.65 (7H, m, NH-CH-CH_2_, CH-Fmoc, CH_2_-Fmoc, N-CH_2_-COOCH_3_, H(5)-cytosine), 7.38 (1H, d, H(6)-cytosine), 7.38-7.90 (8H,m, Fmoc and 5H,m, Bz).

^13^C NMR (CDCl_3_): 47.75 (Fmoc-CH), 49.37 (N-CH_2_-CO), 50.03 (CH-CH_2_-N), 50.42 (NH-CH-CH_2_), 52.79 (CO-CH_2_-cytosine), 67.18 (CH_2_-Fmoc), 67.46 (CH-CH_2_-O), 73.94 (t-butyl, Cq), 97.11 (C(5)-cytosine), 125.63, 127.58, 127.75, 128.12, 128.23, 129.17, 129.47 (Fmoc, Bz), 133.61(C_q_-Bz), 141.77, 144.26 (C_q_-Fmoc), 144.98 (C(6)-cytosine), 150.95 (C(2)-cytosine), 156.75 (COO-Fmoc), 163.18 (CO-CH_2_-cytosine), 168.05 (COO-Bz), 170.09 (COO-OCH_3_).

**Fmoc Ser(tBu)-C(Bz)-OH (10 C)**

Yield: 80% (130 mg)

Mass analysis: m/z (ESI), [M+H]^+^ calculated : 684.0; found :684.2

^1^H NMR (DMSO-d_6_): 1.21 (9H, s, t-butyl-CH_3_), 3.20-4.15 (7H, m, -CH-CH_2_-O, NH-CH-CH_2_, NH-CH-CH_2_, CO-CH_2_-cytosine), 4.21-4.40 (6H, m, CH-Fmoc, CH_2_-Fmoc, NH-CH_2_-COOH, H(5)cytosine), 7.38 (1H, d,H(6)-cytosine), 7.38-8.05 (8H, m, Fmoc, 5H, m, Bz).

^13^C NMR (DMSO-d_6_): 31.22 (t-butyl-CH_3_), 50.73 (CH-Fmoc), 52.07 (CO-CH_2_-cytosine), 53.65 (NH-CH_2_-COOH), 54.23 (NH-CH-CH_2_), 54.90 (NH-CH_2_-CH), 69.45 (CH_2_-Fmoc), 69.59 (CH-CH_2_-O), 76.47 (t-butil-C), 99.72 (C(5)-cytosine), 129.23, 131.05, 131.61, 132.43 (Fmoc, Bz), 136.67 (C_q_-Bz), 147.30, 147.83 (C_q_-Fmoc), 147.90 (C(6)-cytosine), 155.08 (C(2)-cytosine), 159.08 (COO-Fmoc), 159.91 (C(4)-cytosine), 167.34 (COO-CH_2_-cytosine), 171.31 (COO-Bz), 174.27 (COOH).

**Fmoc Ser(OH)-C(Bz)-OH (11 C)**

Mass analysis: m/z (ESI), [M+H]^+^ calculated: 628.0 ; found: 628.1

**^1^H NMR** (DMSO-d_6_)**:** 3.18 - 3.36 (2H, m, CH-CH_2_-N, rotamer B), 3.38 -3.52 (2H, m, CH-CH_2_-N, rotamer A), 3.56 (2H, m, CH-CH_2_-O, rotamer B), 3.61 (2H, m, CH-CH_2_-O, rotamer A), 3.67 (1H, m, NH-CH-CH_2_ rotamer B), 3.79 (1H, m, NH-CH-CH_2_ rotamer A),3.88-4.08 (2H, d, N-CH_2_-COOH), 4.18-4.35 (3H, m, CH-Fmoc, CH_2_-Fmoc), 4.67 (2H, bs, CO-CH_2_-cytosine, rotamer B), 4.88-4.94 (2H, d, CO-CH_2_-cytosine, rotamer A), 6.05 (1H, d, H(5)cytosine), 7.10 (1H, d, NH-CH-CH_2_ rotamer B), 7.23-8.05 (8H, m, Fmoc and 5H, m, Bz), 7.31 (1H, d, NH-CH-CH_2_ rotamer A ), 7.80 (1H, d,H(6)-cytosine)

**^13^C NMR (DMSO):** 50.70 (CH-Fmoc), 51.75 (CO-CH_2_-citosina), 53.68 (NH-CH_2_-COOH), 55.49 (NH-CH-CH_2_), 55.96 (CH-CH_2_-N), 66.77 (CH-CH_2_-O), 69.53 (CH_2_-Fmoc), 99.74 (C(5)-citosina), 129.21, 131.06, 131.60, 132.42 (Fmoc, Bz), 136.67 (C_q_, Bz), 144.71, 147.85 (C_q_ Fmoc), 147.90 (C(6)-cytosine), 155.07 (C(2)-cytosine), 159.02 (CO-Fmoc), 162.06 (C(4)-cytosine), 167.01 (CO-CH_2_-cytosine), 171.31 (COO-Bz), 174.22 (COOH).

**Fmoc Ser(OSO_3_)-C(Bz)-OH (Fmoc-c^S^-OH) (12 C)**

Yield: 91%

Mass analysis: m/z (ESI), [M+H]^+^ calculated ;706.7; found : 706.7

**^1^H NMR** (DMSO-d_6_): 3.30 - 3.49 (2H, m, CH-CH_2_-N, rotamer B), 3.42 – 3.63 (2H, m, CH-CH_2_-N, rotamer A), 3.74 (2H, m, CH-CH_2_-O, rotamer B), 3.83 (2H, m, CH-CH_2_-O, rotamer A), 3.86 (1H, m, NH-CH-CH_2_ rotamer B), 3.88-4.05 (2H, d, N-CH_2_-COOH), 4.00 (1H, m, NH-CH-CH_2_ rotamer A), 4.28 (3H, m, CH-Fmoc, CH_2_-Fmoc), 4.74- 4.67 (2H, d, CO-CH_2_-cytosine, rotamer B), 4.82 - 5.02 (2H, d, CO-CH_2_-cytosine, rotamer A), 6.10 (1H, d, H(5)cytosine), 7.25-8.02 (8H, m, Fmoc and 5H, m, Bz), 7.32 (1H, d, NH-CH-CH_2_ rotamer B),7.56 (1H, d, NH-CH-CH_2_ rotamer A), 7.80 (1H, d,H(6)-cytosine)

**FmocSer(tBu)-G(iBu)-OCH_3_ (8 D)**

Yield: 67% (366 mg)

Mass analysis: m/z (ESI), [M+H]^+^ calculated: 704.9 ; found: 705.0

^1^H NMR (CDCl_3_): 1.14 (6H, d, CH_3_-isobutiryl), 1.17 (9H, s, t-butyl-CH_3_), 2.55-2.75 (1H, m, -CH-(CH_3_)_2_), 3.10-3.32 (2H, m, CH-CH_2_-O), 3.40- 3.58 (2H, m, -CH-CH_2_-N), 3.69 (3H, s, OCH_2_), 3.79 (CO-CH_2_-guanine), 4.18-4.30 (1H, m, NH-CH-CH_2_), 4.38 (1H, m, CH-Fmoc), 4.70-4.80 ( m, 5H, CH_2_-Fmoc, N-CH_2_-COO-OCH_3_, CH-Fmoc), 7.24-7.74 (1H, s, CH-guanine).

^13^C NMR (CDCl_3_)**:** 19.28 (CH_3_-isobutiryl), 27.95 (CH_3_-t-butyl), 36.44 (CH-(CH_3_)_2_-isobutiryl), 47.50 (CH-Fmoc), 47.83 (N-CH_2_-CO), 48.55 (CH-CH_2_-N), 50.01 (NH-CH-CH_2_), 52.78 (OCH_3_), 67.35 (CH_2_-Fmoc), 74.24 (C-t-butyl), 120.53 (C(5)-guanine), 125.33,127.25, 127.84 (Fmoc), 140.28 (C(8)-guanina), 141.78, 144.00 (C_q_ Fmoc), 148.40 (C(2)-guanine), 148.80 (C(8)-guanine), 149.22 (C(4)-guanine), 156.26 (CO-Fmoc), 157.13 (C(6)-guanine), 167.62 (CO-CH_2_-guanine), 170.05 (COOCH_3_), 179.80 (CO-isobutiryl).

**Fmoc Ser(tBu)-G(iBu)-OH (10 D)**

Yield: 81% (290 mg)

Mass analysis: m/z (ESI), [M+H]^+^ calculated : 690.8 ; found: 690.9

^1^H NMR (DMSO-d_6_): 1.13 (6H, d, CH_3_-isobutiryl), 1.19 (9H, s, t-butyl-CH_3_), 2.72-2.80 (1H, m, CH-(CH_3_)_2_), 3.10-3.50 (5H, m, CH-CH_2_-O, CH-CH_2_-N, NH-CH-CH_2_), 4.08 (2H, s, CO-CH_2_-guanine), 4.27- 4.28 (5H, m, CH-Fmoc, CH_2_-Fmoc, N-CH_2_-CO), 7.33-7.87 (8H, m, Fmoc), 7.92 (1H, d, H(8)-guanine).

^13^C NMR (DMSO-d_6_): 22.75 (CH_3_-isobutiryl), 31.20 (CH_3_-t-butyl), 38.69 (CH-(CH_3_)_2_-isobutiryl), 47.99 (CH-Fmoc ), 50.72 (CO-CH_2_-guanine), 52.05 (NH-CH2-CO), 54.32 (CH-CH_2_-N), 55.11 (NH-CH-CH_2_), 69.37 (CH_2_-Fmoc), 69.53 (CH-CH_2_-O), 76.49 (CO-t-butyl), 124.127 (C(6)-guanine), 129.08, 129.17, 131.00, 131.68 (Fmoc), 144.73, 147.75 (C_q_-Fmoc), 147.89 (C(8)-guanine), 151.86 (C(2)-guanine), 153.22 (C(4)-guanine), 158.85 (CO-Fmoc), 159.83 (CO-C(6)-guanine), 171.32 (CO-CH_2_-guanine), 174.72 (COOH), 184 (CO-isobutiryl).

**Fmoc Ser(OH)-G(iBu)-OH (11 D)**

Yield **:** 100%

Mass analysis: m/z (ESI), [M+H]^+^ calculated: 634.9 ; found: 635.2

**^1^H NMR (**DMSO-d_6_)**:** 1.08 (6H, d, CH_3_-isobutiryl), 2.70 (3H, m, CH-(CH_3_)_2_, CH_2_- CH-(CH_3_)_2_), 3.16 - 3.33 (2H, m, CH-CH_2_-N, rotamer B), 3.37- 3.49 (2H, m, CH-CH_2_-N, rotamer A), 3.57 (2H, m, CH-CH_2_-O, rotamer B), 3.67 (2H, m, CH-CH_2_-O, rotamer A), 3.67 (1H, m, NH-CH-CH_2_, rotamer B), 3.90 (1H, m, NH-CH-CH_2_, rotamer A), 3.98-4.07 (2H, d, N-CH_2_-COOH), 4.20- 4.33 (3H, m, CH-Fmoc, CH_2_-Fmoc), 4.95 (2H, bs, CO-CH_2_-guanine, rotamer B), 5.12-5.21 (2H, d, CO-CH_2_-guanine, rotamer A), 7.12 (1H, d, NH-CH-CH_2_ rotamer B), 7.24-7.90 (8H, m, Fmoc), 7.43 (1H, d, NH-CH-CH_2_ rotamer A) , 7.89 (1H, d, H(8)-guanine)

**^13^C NMR (DMSO):** 22.89 ( CH_3_- isobutiryl), 38.70 (CH-(CH_3_)_2_- isobutiryl), 47.99 (CH-Fmoc), 50.69 (CO-CH_2_-guanine), 52.02 (NH-CH_2_-COOH), 53.55 (CH-CH_2_-N), 56.06 (NH-CH-CH_2_), 69.59 (CH_2_-Fmoc), 70.35 (CH-CH_2_-O), 123.30 (C(5)-guanine), 129.14, 131.03, 131.63 (Fmoc), 144.72 (C_q_-Fmoc), 147.81 (C(8)-guanine), 151.95 (C(2)-guanine), 153.22 (C(4)-guanine), 158.76 (CO-Fmoc), 162.42 (CO-C(6)-guanine), 170.73 (CO-CH_2_-guanine), 174.26 (CH-CH_2_-COOH), 184.06 (CO- isobutiryl).

**Fmoc Ser(OSO_3_)-G(iBu)-OH (Fmoc-a^S^-OH) (12 D)**

Yield **:** 100%

Mass analysis: m/z (ESI), [M+H]^+^ calculated: 714.9 ; found : 713.9

**^1^H NMR (**DMSO-d_6_): 1.08 (6H, d, CH_3_-isobutiryl), 2.74 (3H, m, CH-(CH_3_)_2_, CH_2_- CH-(CH_3_)_2_), 3.21 -3.47 (2H, m, CH-CH_2_-N, rotamer B), 3.39 -3.69 (2H, m, CH-CH_2_-N, rotamer A),3.71 (2H, m, CH-CH_2_-O, rotamer B), 3.85 (1H, m, NH-CH-CH_2_, rotamer B),3.87 (2H, m, CH-CH_2_-O, rotamer A), 3.96-4.02 (2H, d, N-CH_2_-COOH), 4.04 (1H, m, NH-CH-CH_2_, rotamer A), 4.22- 4.33 (3H, m, CH-Fmoc, CH_2_-Fmoc), 4.95 (2H, d, CO-CH_2_-guanine, rotamer B),5.14-5.22 (2H, d, CO-CH_2_-guanine, rotamer A), 7.25-7.89 (8H, m, Fmoc), 7.32 (1H, d, NH-CH-CH_2_ rotamer B), 7.53 (1H, d, NH-CH-CH_2_ rotamer A), 7.92 (1H, d, H(8)-guanine)

**Solid phase conditions for coupling of PNA S monomers**

Fmoc Ser(OSO_3_)-T-OH: 50 µL of a 0.3 M solution (7.9 eq.) in an. DMF of monomer, 50 µL of HBTU (0.2 M) (5.2 eq.) in DMF, 50 µL MDCH (0.8 M) in pyridine, 30 minutes.

Fmoc Ser(OSO_3_)-C (Bz)-OH, Fmoc Ser(OSO_3_)-A (Bz)-OH, Fmoc Ser(OSO_3_)-G (iBu)-OH: 50 µL of a 0.3M solution (7.9 eq.) in an. DMF of monomer, 50 µL of HBTU (0.2 M) (5.2 eq.) in DMF, NMM 0.2M, 50 µL of pyridine 0.2M in DMF , 30 minutes
