# Supplementary material for: γ sulphate PNA (PNA S): Highly Selective DNA Binding Molecule Showing Promising Antigene Activity
Source: PLoS One. 2012 May 7;7(5):e35774. doi: 10.1371/journal.pone.0035774 (PMC3346730; doi:10.1371/journal.pone.0035774)
Supplement: Supporting Information S2 — Solid phase synthesis of PNA, PNA S and PNA OH. (DOCX) [file pone.0035774.s005.docx]

**S2 Solid phase synthesis of PNA, PNA S and PNA OH**

Oligomers were obtained by solid phase synthesis on the Fmoc-PAL-PEG-PS resin (0.19 mmol/g) using a 2 µmol scale. The synthesis is carried out by repetitive cycles of deprotection, coupling and capping.

Deprotection: 20% piperidine in DMF, 7 minutes

Capping: Acetic anhydride 5%, 2,6 lutidine 6% in DMF, 5 minutes.

Coupling for PNA S S and PNA OH:

Fmoc-C(Bhoc)-OH: 50 µL of a monomer 0.2 M solution in an. DMF (5 eq.), 50 µL of HATU 0.18 M in DMF (4 eq.), and 50 µL of DIPEA(0.2 M)/ 2,6 lutidine (0.3 M) in DMF 30 minutes.

Fmoc Ser(OSO_3_)-(T)OH (12 A) and Fmoc Ser(OtBu)-(T)OH (10 A): 50 µL of a monomer 0.3 M solution (7.9 eq.) in an. DMF, 50 µL of HBTU (0.2 M) (5.2 eq.) in DMF, 50 µL MDCH (0.8 M) in pyridine, 30 minutes. Modified monomers are always double coupled.

Coupling for PNA: the protocol described by Avitabile et al is followed.

At the end of the synthesis the oligomer is cleaved off the resin and deprotected by treatment with TFA/m-cresol 80/20, 90 min. , r.t.. The TFA is concentrated and the PNA is precipitated with cold diethyl ether. The sample is lyophilized and purified by RP-HPLC using a gradient of acetonitrile (0.1% TFA) in H_2_O (0.1% TFA) from 5 to 25% in 30 minutes.

ESI analysis for PNA S: calculated: [M+H]^+^= 2694.44 m/z ; [M + 2H]^2+^ = 1348.2 m/z, [M + 3H]^3+^= 899.1 m/z

found: [M + 2H]^2+^ = 1346.35 m/z, [M + 3H]^3+^ = 897.15 m/z

ESI analysis for PNA: calculated: [M+H]^+^= 2323.26 m/z; [M + 2H]^2+^ = 1162.63 m/z; [M + 3H]^3+^ = 775.42 m/z

found: [M + 2H]^2+^ = 1163.9 m/z; [M + 3H]^3+^ = 775.96 m/z

ESI analysis for PNA OH: calculated: [M+H]^+^= 2415.44 m/z; [M + 2H]^2+^ = 1208.22 m/z; [M + 3H]^3+^ = 805.74; ; [M + 4H]^4+^= 604.61 m/z; ; [M + 5H]^5+^= 483.88

found: [M + 3H]^3+^ = 804.28 m/z; [M + 4H]^4+^ = 603.20 m/z; [M + 5H]^5+^= 483.90
