# Supplementary material for: γ sulphate PNA (PNA S): Highly Selective DNA Binding Molecule Showing Promising Antigene Activity
Source: PLoS One. 2012 May 7;7(5):e35774. doi: 10.1371/journal.pone.0035774 (PMC3346730; doi:10.1371/journal.pone.0035774)
Supplement: Supporting Information S3 — Solid phase synthesis of FITC labeled PNA and PNA S. (DOCX) [file pone.0035774.s006.docx]

**S3 Solid phase synthesis of FITC labeled PNA and PNA S**

PNA and PNA S were assembled on the Fmoc-PAL-PEG-PS resin (0.19 mmol/g) as described earlier. The Fmoc-Ahx-OH linker was coupled to the N-terminus of PNA S (PNA) using 2.5 eq of Fmoc-Ahx-OH dissolved in a 0.45M HOBT/HBTU solution in DMF (2.49 eq) in the presence of 3.5 eq of NMM at r.t. for 1 hour, two times. Positive Fmoc test confirmed that the reaction was complete. After removal of the Fmoc group by standard procedure, FITC (5 eq) 0.45 M in DMF was coupled in the presence of NMM (7 eq.). The reaction was performed at r.t 90 minutes, two times.

The oligomers were cleaved off the resin, lyophilized, purified by RP-HPLC as described for PNA S .

ESI analysis for FITC- PNA: calculated: [M+H]^+^= 2823.3 m/z; [M + 2H]^2+^ = 1412.6 m/z; [M + 3H]^3+^ = 942.1 m/z; [M + 4H]^4+^ = 706.8 m/z

found: [M + 2H]^2+^ = 1413.2 m/z; [M + 3H]^3+^ = 942.8 m/z; [M + 4H]^4+^ = 707.3 m/z

Characterization by mass spectrometry of FITC- PNA S was tried using several different electrospray instruments and settings, but with no success. We are confident that we isolated the right compound as the UV spectrum, which is shown below together with the HPLC profile of the isolated product, shows the typical absorbance of FITC which is the last molecule coupled on the immobilized oligomer and PNA.
